# Engineered resistance and hypersusceptibility through functional metabolic studies of 100 genes in soybean to its major pathogen, the soybean cyst nematode

**DOI:** 10.1007/s00425-013-1840-1

**Published:** 2013-02-07

**Authors:** Benjamin F. Matthews, Hunter Beard, Margaret H. MacDonald, Sara Kabir, Reham M. Youssef, Parsa Hosseini, Eric Brewer

**Affiliations:** Soybean Genomics and Improvement Laboratory, United States Department of Agriculture, Agricultural Research Service, 10300 Baltimore Ave, Bldg 006, Beltsville, MD 20705 USA

**Keywords:** Soybean, Soybean cyst nematode, Gene expression, Resistance, Transformation, Composite plant

## Abstract

During pathogen attack, the host plant induces genes to ward off the pathogen while the pathogen often produces effector proteins to increase susceptibility of the host. Gene expression studies of syncytia formed in soybean root by soybean cyst nematode (*Heterodera glycines*) identified many genes altered in expression in resistant and susceptible roots. However, it is difficult to assess the role and impact of these genes on resistance using gene expression patterns alone. We selected 100 soybean genes from published microarray studies and individually overexpressed them in soybean roots to determine their impact on cyst nematode development. Nine genes reduced the number of mature females by more than 50 % when overexpressed, including genes encoding ascorbate peroxidase, β-1,4-endoglucanase, short chain dehydrogenase, lipase, DREPP membrane protein, calmodulin, and three proteins of unknown function. One gene encoding a serine hydroxymethyltransferase decreased the number of mature cyst nematode females by 45 % and is located at the *Rhg4* locus. Four genes increased the number of mature cyst nematode females by more than 200 %, while thirteen others increased the number of mature cyst nematode females by more than 150 %. Our data support a role for auxin and ethylene in susceptibility of soybean to cyst nematodes. These studies highlight the contrasting gene sets induced by host and nematode during infection and provide new insights into the interactions between host and pathogen at the molecular level. Overexpression of some of these genes result in a greater decrease in the number of cysts formed than recognized soybean cyst nematode resistance loci.

## Introduction

During pathogen attack, the host plant expresses genes to deter pathogen growth and development (Jones and Dangl 2006; Dodds and Rathjen 2010). Meanwhile, the pathogen often attempts to commandeer the molecular machinery of the plant cell to increase the susceptibility of the host, so it can develop and complete its life cycle. This struggle for dominance results in host-plant gene expression patterns regulated by the host as a defense response, and it also results in host-plant gene expression patterns modulated by the pathogen to enhance its chances of survival. A wide range of phenotypes can be displayed in the host varying from complete resistance through partial resistance to susceptibility. Mechanisms used by some pathogens to break down host resistance are quite complex, as are the responses of the host to repel the attack. Gene expression studies provide insights into the response of the host to infection, but interpretation of these studies is complicated because the pathogen often produces effector molecules in an attempt to modulate host gene expression to provide a more suitable environment for pathogen success.

The interaction of a plant parasitic nematode with the root of a host plant is especially interesting, because the nematode produces a protein-containing cocktail that enters a selected root cell to induce it to form a complex, metabolically active feeding site (Endo 1964). This changes host gene expression patterns. Cyst nematodes, such as the soybean cyst nematode (*Heterodera glycines*; SCN), form a feeding site called a “syncytium.” The life cycle and biology of SCN have been reviewed by Opperman and Bird (1998), Davis et al. (2000), Schmitt et al. (2004), Niblack et al. (2006), Klink et al. (2010b), Gheysen and Mitchum (2011) and others. The secretions of SCN originate from one dorsal and two subventral esophageal secretory glands (Gao et al. 2003). These secretions are injected into a host soybean (*Glycine max*) cell located adjacent to the vascular system of the root to form the feeding site. Numerous proteins are present in the secretion of which many are thought to be effector molecules that inhibit the host plant defense response or promote changes in the host to promote the development of the nematode. Some proteins injected by the nematode into the host cell may be targeted to the nucleus to reorganize transcription (Davis et al. 2000, 2008; Gao et al. 2003; Caillaud et al. 2008; Hogenhout et al. 2009; Haegeman et al. 2011), while others carry out a variety of tasks that help make the host cell more accommodating to the nematode. For example, a cDNA encoding the cellulase, β-1,4-endoglucanase, was isolated from the plant parasitic nematodes SCN and *Globodera rostochiensis* (Smant et al. 1998; Yan et al. 1998), and several other plant cell wall degrading enzymes have also been identified in nematode secretions, including pectate lyase, polygalacturonase, and xylanase. Numerous nematodes produce chorismate mutase (Lambert et al. 1999; Doyle and Lambert 2003; Huang et al. 2005), and enzyme that could affect both salicylic acid (SA) and auxin levels in the host plant. SA is important in triggering portions of the host defense response, while auxin may be involved in feeding site development and maintenance. Superoxide dismutase and proteases were identified in secretions from potato cyst nematode juveniles (Robertson et al. 1999). Many other proteins are also present in these nematode secretions, some with unknown functions. Plant parasitic nematode secretions and parasitism genes have been reviewed in-depth by numerous authors (Davis et al. 2000; Gao et al. 2003; Caillaud et al. 2008; Hogenhout et al. 2009; Haegeman et al. 2011).

The response of soybean to SCN has been examined extensively, because this nematode is recognized to be the major pathogen of soybean in the US. SCN causes an estimated $460 to $818 million per year in losses (Wrather and Koenning 2006, 2009). There are two major genetic sources of resistance to SCN in commonly grown soybean cultivars in the US. One source, PI 88788, provides a slow resistance response to SCN (Klink et al. 2009a). This soybean introduction is the source of resistance to SCN in 85 % of soybean cultivars grown in the US. The other major source of resistance in US-grown soybean cultivars is Peking (PI 548402); it exhibits a rapid response to SCN (Klink et al. 2010a). Neither source alone confers complete resistance to all populations of the nematode. Therefore, new modes of resistance are needed to control SCN.

Resistance in the host can be multigenic, and numerous researchers have studied the genetics of major loci of SCN resistance in soybean (Matson and Williams 1965; Concibido et al. 1994, 2004; Diers and Arelli 1999; Diers et al. 1997; Schuster et al. 2001). For example, several genes (*rhg1, rhg2, rhg3* and *Rhg4*) in soybean cv Peking confer resistance to SCN race 1 (Caldwell et al. 1960; Matson and Williams 1965). The *rhg1* locus confers the most resistance of the known *Rhg* loci, and it reduces by half the number of SCN cysts that form on soybean roots (Concibido et al. 1994, 1997, 2004; Ruben et al. 2006). In PI 437654, resistance to SCN race 3 was mapped to three loci, including the *Rhg4* locus on linkage group A within 1 cM of the *I* locus (Webb et al. 1995). Molecular markers near the *Rhg4* locus have been identified including PCR (Matthews et al. 1998), SSR (Cregan et al. 1999) and SNP (Qui et al. 2003) markers. Recently, candidate genes for both *Rhg1* (Cook et al. 2012; Matsye et al. 2012) and *Rhg4* (Liu et al. 2012) have been identified and tested. However, these genes alone do not provide resistance to all SCN populations. Scientists have been pursuing other approaches to provide resistance to SCN, including transforming plant roots with DNA constructs to silence nematode genes (Bakhetia et al. 2007, 2008; Klink et al. 2009a, b, 2011).

Because there are numerous genes involved in nematode resistance in soybean, a multitude of genes and proteins can be expressed as part of the defense response. In some of the first efforts to study gene expression during the interaction of soybean with SCN, laboratory-manufactured microarrays were used to examine changes in gene expression in soybean roots two days after inoculation (dai) with SCN in a compatible interaction (Khan et al. 2004) and over a period of time, with time points at 6, 12, and 24 hai and 2, 4, 6, and 8 dai (Alkharouf et al. 2004, 2006). Transcripts of genes encoding proline-rich proteins, lipoxygenase, peroxidase, β-1,4-endoglucanase, and stress-induced SAM22 were in high abundance. In a more recent study, soybean roots representing compatible and incompatible interactions with SCN were harvested 12 hai and 3 and 8 dai (Klink et al. 2007a). Genes encoding protease inhibitors, thaumatin-family proteins, cell wall enzymes, lipoxygenase and many other genes were altered in transcript levels. One of the earliest uses of laser capture microdissection was used to isolate RNA from the syncytium, identifying genes induced in their expression in the feeding site (Klink et al. 2005). Microarray gene expression studies of RNA in syncytial cells formed by SCN were also informative in identifying genes expressed by soybean upon attack by SCN. Laser capture microdissection was used by several laboratories to isolate populations of syncytial cells for examination. The compatible and rapid incompatible reactions within syncytial cells of soybean were documented 3, 6, and 9 dai using cv. Peking (Klink et al. 2007b, 2009a) and in the slower incompatible reaction displayed by PI 88788 (Klink et al. 2010a). Gene expression in syncytial cells from the compatible reaction occurring in cv. Williams 82 was also examined at 2, 5, and 10 dai (Ithal et al. 2007). Thousands of genes are up- or down-regulated in these samples. In syncytia of soybean cv Peking, transcripts of genes encoding lipoxygenase, heat shock protein 70 and superoxide dismutase were greatly elevated (Klink et al. 2007b). In syncytia of soybean PI 88788 displaying the resistant interaction, transcripts were elevated for genes encoding enzymes in the jasmonic acid biosynthetic pathway, phenylpropanoid pathway, and suberin biosynthesis (Klink et al. 2010a). Additional studies that incorporated Illumina^®^ deep sequencing of syncytia undergoing the defense response identified some genes that are only expressed during the resistant reaction, but at very high levels (Matsye et al. 2011). The work decreased the large numbers of transcripts typically associated with defense down to several dozen that were induced (Matsye et al. 2011).

The complex interaction of the nematode with its host makes the interpretation of microarray data difficult. The nematode injects effector proteins into the host cell, while the host tries to combat the nematode. It is often uncertain if genes that are up- or down-regulated are controlled by the plant in an effort to provide resistance against the nematode or if host genes have been commandeered by the nematode to enhance susceptibility. Gene expression data, such as that obtained from microarrays, RNA-Seq, and qPCR, are important in studying host–nematode interactions, because genes that are increased and decreased in expression are identified. The data provide new insights into molecular responses and interactions of the host and nematode at the molecular level. However, the data do not indicate if changes in gene expression are the result of the host’s defense response to deter the pathogen, or if modulation of certain genes is due to pathogen effector molecules acting upon gene expression within the host to increase pathogen success. In this report, we provide clear evidence that some soybean genes that are altered in expression during nematode invasion deter nematode growth, while other genes enhance nematode success. We provide new insights into the host–pathogen interaction using a truly functional genomics approach by identifying one group of soybean genes selected from microarray gene expression data that reduce the number of mature female nematodes development on transgenic soybean roots of composite plants when they are overexpressed. We also identify a second group of genes that enhance susceptibility of soybean to SCN when overexpressed, as indicated by the increase in mature female nematodes on those transgenic roots. And we show that a third group of genes do not appear to have a strong effect on susceptibility or resistance to nematodes when overexpressed even though they are altered in expression as indicated by microarray data.

## Materials and methods

### Bioinformatics

One hundred genes were selected from published gene expression studies of the interaction of soybean roots with SCN over time using the Affymetrix microarray platform (Klink et al. 2007a, b, 2009a, 2010a, b; Ithal et al. 2007). The GenBank number of the expressed sequence tag (EST) associated with the gene expression data was used to obtain full-length open reading frames (ORFs) either by building contigs from ESTs found in GenBank or by searching the database using the DNA or predicted protein sequence against GenBank or searching the soybean genome database found at Phytozome.net (Joint Genome Institute, USDOE; Center for Integrative Genomics, UC Berkeley). Primers for PCR amplification of the ORF were designed using Primer 3 (http://biotools.umassmed.edu/bioapps/primer3_www.cgi) and OligoAnalyzer 3.1 (Integrated DNA Technologies, Coralville, IA, USA). For promoter analysis, DNA sequences 2,000 nt upstream of the ATG start site of selected genes were obtained from the *G. max* genome database found at Phytozome.net and examined for the presence of the auxin response element TGTCTC.

### Amplification and cloning of ORFs

The ORFs of target genes were cloned using the Gateway^®^ (Invitrogen, Carlsbad, CA, USA) system. The ORFs were amplified from template cDNA using cDNA libraries previously reported (Heinz et al. 1998; Khan et al. 2004), representing RNA from the SCN-resistant soybean cultivar Peking (PI 548402) 3 days after infection (dai) with SCN NL1-RHp (Hg Type 7; race 3). The UniZap Library (Heinz et al. 1998) was made from roots and shoots of soybean cultivar *Glycine max* Peking 2–3 dai displaying the resistant interaction with SCN NH1-RHp. The TriplExZ library Khan et al. 2004) was made from roots only of the soybean cultivar *Glycine max* Peking 2–4 dai displaying the resistant interaction with SCN NH1-RHp. The ORFs were amplified using gene-specific PCR primers containing CACC at the 5′ end of the forward primer, which is necessary for directional cloning using the Gateway^®^ (Invitrogen) system.

**Fig. 1:**
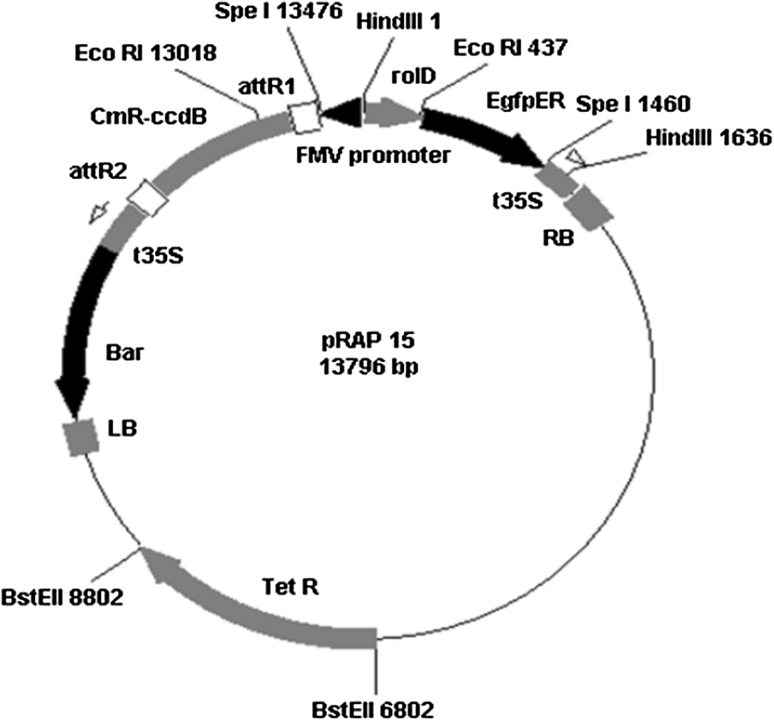
The gene expression vector pRAP15 is designed to overexpress genes using the figwort mosaic virus (FMV) promoter. The vector contains the gene encoding enhanced green fluorescent protein (eGFP) driven by the *Agrobacterium rhizogenes* *rol*D promoter so transformed roots can be identified easily. It also contains the attR1 and attR2 sites for Gateway^®^ cloning, a tetracycline resistance gene (TetR) for bacterial selection, and the *bar* gene for chemical selection of transformed plant cells using the herbicide BASTA^®^

The PCR amplicons were cloned into pENTR using a pENTR™ Directional TOPO^®^ Cloning Kit (Invitrogen) and transformed into competent *Escherichia coli* cells using One Shot^®^ Mach1™ T-1 chemically competent cells (Invitrogen), and transformed clones were selected with 50 μg mL^−1^ kanamycin. The sequence of each insert was confirmed by DNA sequencing using the vector-specific primers M13-F and M13-R (Table 1). The inserts were transferred to the gene expression vector pRAP15 (Fig. 1; Matsye et al. 2012) at the attR1 and attR2 sites designed for directional cloning using Invitrogen’s Gateway^®^ technology mediated by LR Clonase™ II Enzyme Mix (Invitrogen). In this vector, the figwort mosaic virus (FMV) promoter drives expression of the inserted gene. To enable visualization of transformed roots, the vector contains the gene encoding enhanced green fluorescent protein gene (eGFP; Haseloff et al. 1997) controlled by the *rol*D promoter that gives strong root expression. The gene encoding tetracycline resistance (TetR) provides antibiotic selection. The Clonase II reaction product was used to transform *E. coli* cells as described above with selection on 10 μg mL^−1^ tetracycline plates. Presence of the insert in the correct orientation downstream from the FMV promoter was confirmed by PCR using the FMV-specific primer FMV-F (Table 1) and the *G. max* gene-specific reverse primer. The pRAP15 vector bearing the inserted gene of interest was used to transform chemically competent *Agrobacterium rhizogenes* ‘K599′ cells (Haas et al. 1995) using the freeze–thaw method (Hofgen and Willmitzer 1988) with selection on 5 μg mL^−1^ tetracycline plates. Presence of the insert in pRAP15 was confirmed as described above. Presence of the gene encoding eGFP was confirmed by PCR using eGFP-F and eGFP-R primers (Table 1) and eGFP was confirmed visually in transgenic roots. Presence of the *A.* *rhizogenes* R_i_ plasmid, which is necessary for root transformation, was confirmed by PCR using R_i_-F and R_i_-R primers (Table 1).

**Table 1:** Primers used in PCR amplification and sequencing

Primer	Sequence	Amplicon size (bp)
M13-F	5′-GTAAAACGACGGCCAG-3′	–
M13-R	5′-CAGGAAACAGCTATGAC-3′	
FMV-F	5′-AAGAAGCCCTCCAGCTTCAAAG-3′	
eGFP-F	5′-ATGGTGAGCAAGGGCGAGGAGC-3′	706
eGFP-R	5′-TCGTCCATGCCGAGAGTGATCCCG-3′	
R_i_-F	5′-TCAGCCTCCCCGCCGGATG-3′	812
R_i_-R	5′-ATGCAAAAGACAGGATTGATCGCA-3′

### Formation of composite soybean plants

Composite soybean plants were made that contained untransformed shoots and transformed roots as described previously (Klink et al. 2009b; Ibrahim et al. 2011). *A. rhizogenes* clones containing the genes of interest were grown as described previously Klink et al. 2009b; Ibrahim et al. 2011). *A. rhizogenes* containing pRAP15 with no gene of interest were grown to transform control roots. Briefly, for each gene tested, 100 soybean cv. Williams 82 PI518671 plants were grown in Promix in the greenhouse. At approximately 7 days, the plantlets were cut at the soil line, and the base of each plant was submerged in co-cultivation medium containing a suspension of *A. rhizogenes* with an OD_600_ of 0.5. The co-cultivation solution was composed of 4.4 mg/mL MS salts (Duchefa Biochemie) and 3 % sucrose at pH 5.7. After vacuum infiltration for 30 min, the plantlets were co-cultivated on a rotary shaker overnight at 23 °C at 65 rpm. The stems were rinsed with water, placed in a beaker of water, and incubated for approximately 48 h at 23 °C in a growth chamber. The plantlets were planted in pre-wetted Promix in the greenhouse. Four weeks later, non-transformed roots were excised, while transformed roots were recognized by the presence of eGFP and retained. Fluorescence of eGFP was perceived using a Dark Reader Spot lamp (Clare Chemical Research, Dolores, CO, USA). Non-transformed roots were removed again 2 weeks later.

### Nematode preparation and assay

SCN population NL1-RHg was grown in a greenhouse at the United States Department of Agriculture, Beltsville, MD as described previously (Klink et al. 2007a). Briefly, mature SCN females and cysts were washed from roots of susceptible soybean plants 3 months after inoculation and purified by sucrose flotation (Jenkins 1964). The purified females and cysts were placed on a 3-inch diameter, 150 μm sieve (Newark Wire Cloth Co, Clifton, NJ, USA), partially submerged in a small tray of water. Females and cysts were gently crushed with a rubber stopper against the sieve, allowing collection of eggs in the tray below. The eggs were purified further by passing the suspension through a 61 μm sieve. Eggs were collected on a 25-μm sieve that allowed small particles to pass. To reduce microbial contamination, a 0.5 % sodium hypochlorite solution was poured into the sieve and slowly drained during 1.5 min before washing the sieve with one liter of sterile double distilled H_2_O. Eggs were placed in 120 mL of sterile 3 mM ZnSO_4_ in a small, covered tray and allowed to hatch on a rotary shaker at 25 rpm at 26 °C. Four days later, SCNs at the J2 stage were separated from unhatched eggs by passing the solution through a 30 um mesh nylon cloth (Spectrum Labs Inc, Rancho Dominguez, CA, USA). J2s were concentrated by placing 200 mL of the solution in 1-L glass beakers on a rotary shaker at 100 rpm. J2s quickly gathered to the center bottom of the beaker and were collected with a Pasteur pipette. Volume of the solution was adjusted with sterile water to achieve a concentration of 1,000 J2/mL for inoculation of transgenic roots of composite plants.

Twelve to twenty transformed composite plants were used in each assay. Two holes, 4-cm deep, were made in the sand on either side of the plant. One millilitre of nematode inoculum was added to each hole to provide 2,000 juveniles per plant. After 35 days, test plants were placed in water and the roots were gently rubbed to dislodge the female nematodes. These were collected between nested 850- and 250-μm sieves and washed onto lined filter paper in a Buchner funnel (Krusberg et al. 1994). Females were counted under a dissecting microscope.

### qRT-PCR

The expression level of two genes transformed into soybean roots was confirmed by qRT-PCR as described previously (Tremblay et al. 2010; Ibrahim et al. 2011). The genes were a GDSL esterase/lipase (Lab ID: C45; GenBank ID: NP_001242371.1) and a possible lysine decarboxylase (Lab ID: C49; Phytozome ID: Glyma17g37660.1). Three individual soybean roots were harvested per construct. RNA was extracted using a Qiagen RNeasy Mini Kit according to the manufacturer’s instructions. Contaminating DNA was removed by DNase digestion using a TURBO DNA-free kit (Invitrogen) according to the manufacturer’s instructions. RNA was converted into a cDNA library using the Superscript III First-Strand Synthesis System for RT-PCR (Invitrogen) according to manufacturer’s instructions. Soybean roots transformed with pRAP15 served as controls to measure endogenous gene expression. Primers were designed to produce an amplicon between 100 and 200 bp (Table 2). The gene encoding rs-21 (Glyma09g00210.1) served as a positive control (Klink et al. 2005). Lambda phage DNA served as the standard for quantification. Reactions containing no RNA or template processed with no reverse transcriptase were used as negative controls. qRT-PCR reactions were conducted in triplicate for each root cDNA sample using the Brilliant II Syber Green Master Mix qPCR Kit (Stratagene, La Jolla, CA, USA) according to the manufacturer’s instructions. Relative levels of gene expression were determined using the Stratagene Mx3000P Real-Time PCR system (Stratagene) as described by the manufacturer. The SYBR green dissociation curve of the amplified products demonstrated the production of only one product of the expected size per reaction. Data analysis was performed according to the sigmoidal model (Rutledge and Stewart 2008) to get absolute quantification as described in Tremblay et al. (2010).

**Table 2:** Primers used in qRT-PCR assays

Clone ID	Forward primer	Reverse primer	Amplicon size (bp)
Rs-21	5′-CTAAGATGCAGAACGAGGAAGG-3′	5′-GAGAGCAAAAGTGGAGAAATGG-3′	168
C45	5′-GCAGATGGGTTAATGGAGCTTTGTG-3′	5′-GACATCCAATGCAGACTAGGTTTCC-3′	203
C49	5′-CGTGGATGGGTACTACAACTCGTTG-3′	5′-TGGTTCATCTCCCAACTTTGCTTTG-3′	186

### Statistical analysis

Outliers in the female count data were removed using Grubbs’ test (Grubbs 1969) at the GraphPad QuickCalcs Web site (http://graphpad.com/quickcalcs/grubbs1/). Normality of the data was checked using the Shapiro–Wilk test (Shapiro and Wilk 1965; online version implemented by S. Dittami, http://dittami.gmxhome.de/shapiro/). Means were compared using Welch’s unpaired *t* test for unequal variance (Welch 1947) at the GraphPad QuickCalcs Web site (http://graphpad.com/quickcalcs/ttest1/). The female index was calculated as follows: Female index = (*N*_g_/*N*_c_) × 100, where *N*_g_ = mean number of females for the gene of interest and *N*_c_ = mean number of females for the empty pRAP15 control. In some previously reported studies, the FI was calculated from a total of 3–10 experimental and 3–10 control plants with each plant serving as a replicate while experimental replicates may or may not be performed (Golden et al. 1970; Riggs and Schmitt 1988, 1991; Kim et al. 1998; Niblack et al. 2002). In the presented analysis, the number of experimental plants met or exceeded investigations testing SCN infection in genetically engineered soybean (Steeves et al. 2006; McLean et al. 2007; Mazarei et al. 2007; Li et al. 2010; Melito et al. 2010; Ibrahim et al. 2011). In our experiments, eight or more plants were examined for each gene and each control. We started with 105 genes that were tested. However, we eliminated five gene tests; two had non-normal distributions, and three were omitted due to low number of plants because of poor plant health.

## Results

### Gene selection and assay system

One hundred soybean genes were cloned, sequenced, and overexpressed in roots of a soybean cultivar susceptible to SCN, Williams 82 (PI 518671) to determine their effect on SCN development (Table 3). The genes were chosen from gene expression data derived from microarray experiments reported previously (Klink et al. 2007a, b, 2009a; Ithal et al. 2007). Microarray data from Klink et al. (2007a, b) represent gene expression in soybean cv. Peking roots and in the syncytium, respectively, during the compatible (SCN population TN8) and incompatible (SCN population NL1-Rhg) interactions; microarray data from Klink et al. (2009a) define gene expression from laser microdissected syncytia from roots of cv. Peking using the same nematode populations; microarray data from Klink et al. (2010a) define gene expression from laser microdissected syncytia from roots of soybean PI 88788 using SCN NL1-Rhg providing the incompatible interaction. The ORF of each gene was cloned into the pRAP15 vector (Fig. 1) for overexpression in soybean roots.

**Table 3:** Hundred soybean genes cloned

Phytozome	Function	Gene ID	OE gene mean # females	*n*	SEM	Control mean # females	*n*	SEM	FI (% of control)	*P* value
Glyma12g07780.3	Ascorbate peroxidase	**C21**	15	12	2	58	24	6	**26**	**<0.0001**
Glyma08g02610.1^a^	β-glucanase	**A12**	49	21	7	127	41	9	**39**	**<0.0001**
Glyma19g38390.1	Momilactone A synthase-like	**A24**	24	10	5	58	24	6	**41**	**0.0001**
Glyma07g05830.2	Unknown, cytoch b5-like	**C19**	24	11	6	58	24	6	**41**	**0.0006**
Glyma08g14550.1^a^	Lipase	**A25**	35	18	6	84	41	8	**42**	**<0.0001**
Glyma15g16560.1^a^	DREPP membrane	**R08**	27	24	4	56	32	7	**48**	**0.0005**
Glyma13g22650.1^a^	Unknown, plastocyanin-like	**A30**	29	22	5	60	23	9	**48**	**0.006**
Glyma10g30340.1	Unknown	**A08**	62	10	9	127	21	13	**49**	**0.0003**
Glyma19g19680.1	Calmodulin SCaM-3	**S01**	64	10	9	127	21	13	**50**	**0.0004**
Glyma02g46040.1	OTU-like cysteine protease	**M10**	28	15	3	53	17	9	**53**	**0.017**
Glyma08g11490.1	Serinehydroxymethyltransferase 2	**A35**	70	10	9	127	21	13	**55**	**0.0009**
Glyma05g38130.1	Thaumatin	**A07**	71	10	8	127	21	13	**56**	**0.0007**
Glyma20g24280.1	NADH:ubiquinone oxidoreductase	C17	94	8	23	167	8	34	56	0.10
Glyma14g00640.1	Chlorophyll A-B binding protein	**C40**	96	18	13	159	23	15	**60**	**0.003**
Glyma03g32850.1	HSP70	**C14**	36	11	5	58	24	6	**62**	**0.007**
Glyma04g10880.1	Phosphate responsive	**C28**	100	24	14	159	23	15	**63**	**0.005**
Glyma08g05710.1^a^	HMG I/Y	**C16**	123	29	13	192	29	17	**64**	**0.002**
Glyma13g29690.1	Aquaporin	**A43**	53	9	7	82	10	10	**65**	**0.03**
Glyma20g24810.1	Cinnamate 4-hydroxylase	**A11**	83	10	11	127	21	13	**65**	**0.015**
Glyma04g08520.1	Transporter	R48	27	10	5	41	23	5	66	0.06
Glyma13g27020.1	Annexin	C12	110	7	32	167	8	34	66	0.25
Glyma13g23680.1	Nitrate/oligopeptide transporter	C43	43	12	8	63	16	9	68	0.09
Glyma20g27950.1	Polyubiquitin	**C37**	109	21	14	159	23	15	**69**	**0.02**
Glyma07g30880.1	Monosaccharide transporter	**A10**	89	10	13	127	21	13	**70**	**0.047**
Glyma06g41610.1	Thioredoxin-related	**C42**	143	25	12	201	21	19	**71**	**0.015**
Glyma10g00970.1	Unknown	A44	43	9	7	59	14	6	73	0.13
Glyma09g33730.1	JAI1-like TF	G68	49	11	8	65	13	8	75	0.18
Glyma19g02180.1	AAA + -type ATPase	C13	126	9	41	167	8	34	75	0.45
Glyma09g33140.1	Dirigent-like protein	A15	28	12	6	37	14	6	76	0.29
Glyma12g05840.1	Lipoxygenase	G91	50	8	4	65	13	8	77	0.11
Glyma17g05770.1	NADH:flavin oxidoreductase	G55	95	10	16	121	23	17	79	0.29
Glyma14g09990.1^a^	Phytosulfokine precursor protein	A27	66	22	10	84	41	8	79	0.17
Glyma16g07830.1	2OG-Fe(II) oxygenase	C52	46	12	8	58	24	6	79	0.22
Glyma08g11520.1	Chalcone synthase	A52	47	11	12	59	14	6	80	0.41
Glyma11g10240.4^a^	Expansin; rare lipoprotein A	C27	140	8	22	167	8	34	84	0.52
Glyma04g40580	O-methyltransferase	A42	107	10	17	127	21	13	84	0.35
Glyma17g03130.1	Epoxide hydrolase	A09	108	10	18	127	21	13	85	0.41
Glyma06g14960.1	Superoxide dismutase	C07	54	12	7	63	16	9	86	0.42
Glyma20g36700.1	Unknown	A51	109	10	14	127	21	13	86	0.34
Glyma17g06220.1	Cytokinin dehydrogenase	A37	32	11	8	37	14	6	86	0.59
Glyma08g41040.1	Unknown, possible TF	A38	76	12	16	87	16	9	87	0.56
Glyma05g30380.1^a^	Cu binding	A22	58	18	7	65	18	6	89	0.41
Glyma14g38220.2	DAD1	R05	37	10	4	41	23	5	90	0.57
Glyma17g37660.1	Lysine decarboxylase	C49	146	18	20	159	23	15	92	0.61
Glyma0466s00200.1	GDSL esterase/lipase	C45	147	21	12	159	23	15	92	0.53
Glyma20g10960.1	Cdc2-related protein kinase	M06	50	14	11	53	17	9	94	0.85
Glyma19g36620.1	Phenylalanine ammonia-lyase	A53	120	10	20	127	21	13	94	0.76
Glyma08g21420.1	Acid phosphatase	C20	195	10	35	201	21	19	97	0.87
Glyma08g25950.1	Cytochrome P450	C23	57	12	11	58	24	6	98	0.95
Glyma13g26960.1	Annexin	A60	136	12	13	136	19	16	100	0.99
Glyma20g36790.1	Auxin repressor	R24	59	12	10	59	14	6	101	0.98
Glyma09g05230.1	DREPP membrane polypeptide	C53	160	21	22	159	23	15	101	0.97
Glyma03g38520.1	Cysteine proteinase, Cathepsin L	M08	55	14	8	53	17	9	104	0.89
Glyma17g07190.1	4-coumarate:coenzyme A ligase	A48	67	11	10	63	16	9	106	0.79
Glyma13g16620.1	Nuclease HARBI1-like	A49	63	9	17	59	14	6	107	0.84
Glyma16g27350.1	Sucrose transport	A68	88	10	19	82	10	10	107	0.80
Glyma06g47740.1	Pectin esterase inhibitor	A20	65	12	9	59	19	5	110	0.51
Glyma02g47940.1	Phenylalanine ammonia-lyase	A45	65	11	15	59	14	6	111	0.72
Glyma12g01580.1	Heat shock protein	A39	143	10	14	127	21	13	113	0.42
Glyma03g37940.1	WRKY TF	C34	67	10	11	59	19	5	114	0.48
Glyma02g38030.1	TFIIA	C39	230	10	28	201	21	19	114	0.42
Glyma20g23080.1	Calreticulin	C36	70	10	9	59	19	5	119	0.28
Glyma02g33780.1	Glutathione S-transferase	A33	162	12	17	136	19	16	119	0.28
Glyma20g30720.1	Abscissic stress	R03	49	10	6	41	23	5	120	0.31
Glyma08g04740.1	Unknown	C03	244	10	22	201	21	19	121	0.16
Glyma20g35270.1	Auxin-responsive	R07	50	10	7	41	23	5	122	0.28
Glyma04g41750.1	Ubiquitin conjugating enzyme	C15	206	7	45	167	8	34	123	0.51
Glyma01g39460.1	O-methyltransferase	A03	73	11	10	59	19	5	124	0.22
Glyma06g12340.1	ACC oxidase	R28	51	9	7	41	23	5	124	0.27
Glyma02g48010.1	Membrane-associated ring finger	M40	66	15	14	53	17	9	125	0.46
Glyma02g16480.1	Kelch repeat, F-box	R27	53	10	8	41	23	5	129	0.22
Glyma15g14040.1	Berberine-like	A31	48	12	7	37	14	6	130	0.31
Glyma11g10240.1	Pollen allergen	A64	177	11	19	136	19	16	130	0.12
Glyma03g27740.1^a^	Cytochrome P450	A13	66	24	8	50	33	4	132	**0.06**
Glyma11g37370.1	B12D protein	A26	77	12	11	58	24	6	133	0.14
Glyma17g01230.1	BAF60 TF	A23	78	11	13	58	24	6	134	0.19
Glyma12g33530.1	Phi-1 fasciclin-like	V01	169	20	24	129	25	17	132	0.17
Glyma13g23400.1	Ribosomal protein S11	C06	228	19	23	165	19	22	138	**0.053**
Glyma04g04310.1	WOX TF	A06	189	11	24	136	19	16	139	**0.08**
Glyma13g30950.1	Unknown	**A05**	84	24	9	59	19	5	**142**	**0.01**
Glyma01g42670.1	Thaumatin PR5b	R04	289	10	51	201	21	19	144	0.14
Glyma13g01230.1	PR1-like	**A36**	119	11	11	82	10	10	**145**	**0.03**
Glyma08g27590.1	Membrane type III	**A34**	120	7	14	82	10	10	**146**	**0.03**
Glyma20g26610.1	Secretory protein	**A50**	96	12	12	63	16	9	**152**	**0.04**
Glyma05g36830.2	Lipase	M57	81	14	11	53	17	9	153	**0.07**
Glyma04g08200.1	Endopeptidase	**C08**	255	15	38	165	19	22	**155**	**0.049**
Glyma17g03360.1	SAM22 PR10	**A01**	94	12	10	59	19	5	**159**	**0.006**
Glyma02g40000.1	Cationic peroxidase	**A02**	94	12	13	59	19	5	**159**	**0.025**
Glyma02g42290.1	Auxin permease	**A18**	95	12	13	59	19	5	**161**	**0.02**
Glyma13g44700.1	Cinnamoyl CoA reductase	**A46**	221	12	26	136	19	16	**162**	**0.01**
Glyma09g31110.1	Metal ion transport	**C55**	97	12	10	59	19	5	**164**	**0.003**
Glyma17g35360.1	Unknown, rsbQ-like	C29	188	12	39	114	10	24	165	0.13
Glyma07g16420.1	Unknown	**R25**	69	10	10	41	23	5	**168**	**0.02**
Glyma04g39860.1	Peroxidase III	**A04**	115	12	11	59	19	5	**195**	**0.0003**
Glyma15g41840.1	Lipase	**K01**	266	12	35	136	19	16	**195**	**0.004**
Glyma17g13550.1	Pectate lyase	**A32**	73	10	14	37	14	6	**197**	**0.04**
Glyma19g09810.1	Cupin domain	**A61**	291	11	34	136	19	16	**214**	**0.001**
Glyma15g03390.1	Unknown	**A21**	135	12	16	59	19	5	**229**	**0.0006**
Glyma02g37020.1	UDP-glucuronate 4-epimerase	**C32**	146	12	18	63	16	9	**232**	**0.0007**
Glyma16g33840.1	Oligopeptide transporter	**R30**	104	10	13	41	23	5	**254**	**0.001**

Genes with the Phytozome numbers are provided in above table. Each clones was given a unique gene identification number; the predicted function of the gene was determined using Phytozome annotations, blastp, and searches of other databases when necessary. The mean number of mature female nematodes collected from transformed roots containing the gene and the mean of those collected from control roots transformed with empty vector are given. The number of plants (*n*) and standard error of the mean (SEM) is presented for each. The effect of overexpression of each gene on the number of SCN females that developed on the transformed roots was determined and is expressed as the female index (FI) percent of control values with *P* value (confidence interval). The Gene ID, FI and *P* value of genes with a *P* value of < 0.05 are in bold

^a^Trials that were repeated and combined

An assay system was devised using composite soybean plants having only roots that were transformed with the gene construct, because assays could be conducted more quickly than if whole transgenic soybean plants were used. Transformed roots were visualized by the presence of eGFP, non-transformed roots were excised from the composite plant, and the transgenic roots were inoculated with SCN J2 juveniles. Plants were harvested 35 dai, and mature SCN females were collected and counted. The female index was calculated using eight or more composite plants with roots recognized as transgenic due to the presence of eGFP.

**Fig. 2:**
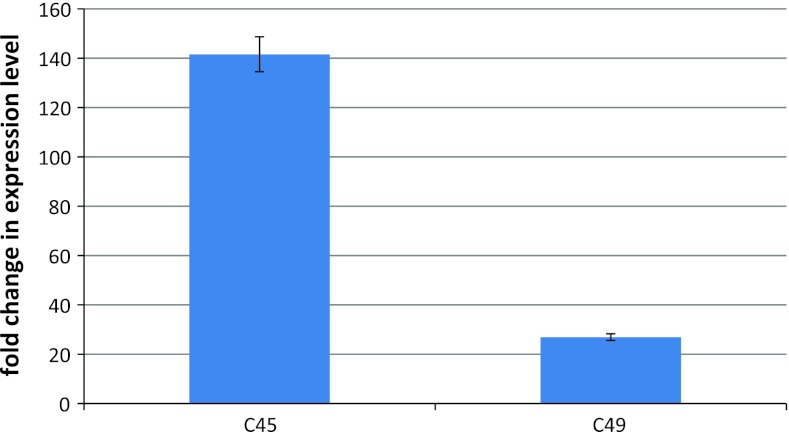
Expression levels of transcripts of genes encoded by C45 and C49 as measured by qRT-PCR in transformed roots (*n* = 3)

To confirm that transcripts of the genes were overexpressed, transcript levels from three roots for each of two constructs, C45 and C49, were measured using qRT-PCR. The transcript levels of genes encoded by C45 and C49 were increased 141- and 27-fold, respectively, as compared to control roots transformed with empty pRAP15 (Fig. 2).

### Genes decreasing the female index of SCN by 50 % or more

**Fig. 3:**
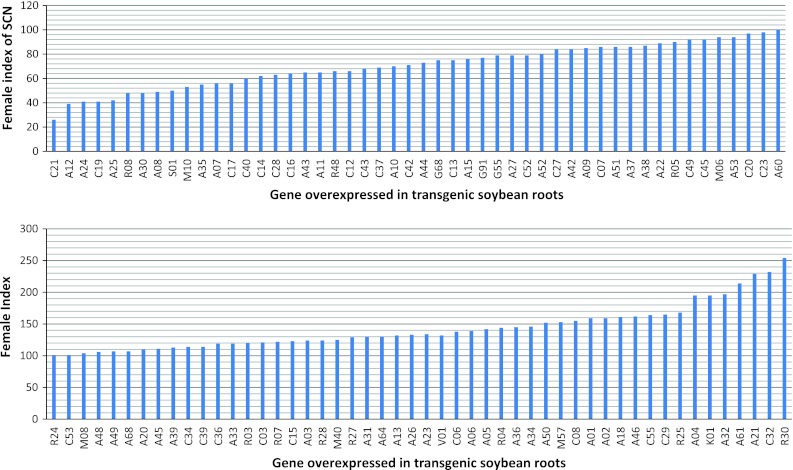
Female index of SCN obtained from assays of transgenic roots of composite soybean plants overexpressing soybean genes

Nine genes decreased the Female Index by 50 % or more when overexpressed in transgenic roots (Fig. 3; Table 3). Gene C21 encodes ascorbate peroxidase 2; gene A12 encodes a β-1,4-endoglucanase. Gene A24 encodes a momilactone A synthase-like protein, while gene R08 encodes a DREPP membrane protein. Two genes may be involved in signaling: a lipase (A25) and calmodulin (S01). The three remaining genes are of unknown function, including a gene encoding a protein similar to proteins in the cytochrome b5 family (C19), a plastocyanin-like protein (A30), and A08.

Overexpression of the gene encoding ascorbate peroxidase 2 (C21; Glyma12g07780.3) decreased the SCN female index by more than 70 %. The encoded protein is 250 aa, and it has one closely related homolog, Glyma11g15680.2 (93 %) at the aa level. Microarray analysis of transcript levels indicate that it was unchanged in the incompatible interaction of Peking with SCN at 3, 6 and 9 dai, but was increased 77-, 40-, and 50-fold in the incompatible interaction of PI88788 with SCN at the same time points (Klink et al. 2007a, b, 2010a, 2011). Klink et al. (2011) showed that there are differences in gene transcript abundance during the incompatible interaction of Peking and PI 88788 with SCN. No change in the level of transcripts of this gene was found in the susceptible interaction of Williams 82 with SCN (Ithal et al. 2007).

Gene A12 encodes a β-1,4-endoglucanase (Glyma08g02610.1; EST BI943300) that catalyzes the hydrolysis of cellulose. The 1875 bp ORF encodes a 625 aa protein. Overexpression of gene A12 decreased the female index to 39 % of the control value. There are fifteen homologs (>2e-34) of this β-glucanase in soybean with Glyma05g36930.1 being most closely related (97.8 % nt similarity). Transcripts of this gene were elevated over 300-fold in syncytia formed by SCN during the incompatible interaction with Peking at 3 dai, over 200-fold at 6 dai and over, and 100-fold at 9 dai (Klink et al. 2007a, b), while the levels of transcripts of this gene were unchanged in syncytia analyzed from the compatible interaction of SCN with Williams 82 (Ithal et al. 2007).

Overexpression of gene A24 (Glyma19g38390.1) decreased the female index to 41 % of the control. The gene encodes a 278 aa protein that is a perfect match at the aa level with a computer-predicted soybean momilactone A synthase-like protein (XP_003554453.1). The protein contains a conserved domain similar to that found in secoisolariciresinol dehydrogenase-like proteins, which are functionally diverse family of oxidoreductases. The protein has high similarity with Glyma 03g35760, Glyma 11g18570, Glyma12g09780, Glyma15g27630, and Glyma03g26590. Transcript abundance was increased 220-fold at 3 dai, 48-fold at 6 dai, and 21-fold at 9 dai in syncytia from the incompatible interaction of SCN with Peking (Klink et al. 2007a, b, 2009a, b) and 66-fold at 2 dai, 38-fold at 5 dai, 10-fold increase at 10 dai in syncytia from the compatible interaction of SCN with Williams 82 (Ithal et al. 2007).

Overexpression of gene C19 (Glyma07g05830.2; EST CF807399) also decreased the female index to 41 % of the control. It is a member of the cytochrome b5 superfamily, and its function is unknown. It has numerous homologs with high similarity at the aa level. It has 97.2 % aa similarity with Glyma16g02410.1, 76.8 % aa similarity with Glyma04g41010, 76.1 % aa similarity with Glyma06g13840.1, 71.8 % aa similarity with Glyma03g42070, and 71.1 % aa similarity with Glyma19g44780. It was approximately 2-fold induced in syncytia from the incompatible interaction of SCN with Peking at 3, 6, and 9 dai (Klink et al. 2007a, b, 2009a) and in syncytia from the compatible interaction at 2, 5, and 10 dai with Williams 82 (Ithal et al. 2007).

When gene A25 was overexpressed in transgenic soybean roots, the female index decreased to 42 % of the control. A25 encodes a lipoxygenase/lipase (Glyma08g14550.1; EST CD409280), a member of the PLAT-domain family. It has 92 % aa similarity with Glyma05g31310.1 and 86.3 % aa similarity with Glyma11g38220.1. Transcripts of this lipoxygenase were elevated over 250-, 100-, and 60-fold in syncytia displaying the incompatible interaction of SCN with Peking at 3, 6, and 9 dai, respectively (Klink et al. 2007a, b, 2009a).

A DREPP membrane protein-family member composed of 207 aa is encoded by gene R08 (Glyma09g05230.1). When it is overexpressed in soybean roots, the SCN female index was 48 % of control. It has complete aa identity with NP 001237575.1. It has only one closely related homolog, Glyma15g16560.1. The abundance of transcripts encoding this gene was decreased −23-fold at 2 dai, −3.6-fold at 5 dai, and −20-fold at 10 dai in syncytia from the compatible interaction of SCN with soybean roots (Ithal et al. 2007), while no change in transcript abundance was detected in the incompatible interaction at any of the time points measured by Klink et al. (2007a, b, 2009a, b).

Gene A30 (Glyma13g22650; EST BE659015) overexpression reduced the female index to 48 % of the control. It encodes a 336 aa plastocyanin-like protein of unknown function. Its closest homolog is Glyma17g12150.1 at 42.3 % aa similarity. Transcripts of gene A30 were elevated approximately 63-, 120-, and 57-fold in syncytia from the incompatible interaction of SCN with Peking at 3, 6, and 9 dai, respectively (Klink et al. 2007a, b, 2009a), while its transcripts were elevated 10-, 6-, and 5-fold in syncytia from the compatible interaction of SCN with Williams 82 at 2, 5, and 10 dai, respectively (Ithal et al. 2007).

Overexpression of gene A08 (Glyma10g30340.1; EST CF805971) reduced the female index to 49 % of the control. It is of unknown function and had no significant matches in Pfam, but it has been identified as an uncharacterized protein in other plants such as *Ricinus communis*, *Populus trichocarpa*, and *Medicago truncatula*, according to our blastp results. It has one close relative, Glyma20g36700. Transcript levels were unchanged in syncytia from incompatible and compatible interactions of SCN with Peking and Williams 82, respectively, as compared to control cells (Klink et al. 2007a, b, 2009a; Ithal et al. 2007). However, gene A08 is of unknown function; its transcripts are increased in abundance 12-fold in roots infected by the root-knot nematode (RKN; *Meloidogyne incognita*) 12 dai and 26-fold 2 months after infection (Ibrahim et al. 2011).

The gene S01, encoding calmodulin-2 (Glyma19g19680.1; EST L01432.1) reduced the female index of SCN 50 % when overexpressed. Several soybean genes encode closely-related proteins, including Glyma02g44350.1, Glyma14g04460.1, and Glyma05g12900.1, all had 100 % similarity at the aa level. Transcript levels of this gene were unchanged in syncytia from the incompatible interaction of SCN with Peking (Klink et al. 2007a, b). However, transcript levels were elevated approximately 2-fold in syncytia from the compatible reaction of SCN with Williams 82 at 2 and 5 dai, and they were fivefold elevated at 10 dai (Ithal et al. 2007).

Overexpression of gene A35 decreased the female index 55 % of the control, and it is of interest, because it encodes a serine hydroxymethyltransferase located on chromosome eight (Glyma08g11490) of the soybean genome at the *Rhg4* locus (Matson and Williams 1965) encoding a resistance gene for SCN (Cregan et al. 1999; Lewers et al. 2002; Liu et al. 2012). Microarray analysis of the expression of this gene indicates that the gene is increased in expression 3.1-fold at 2 dai and 2.1-fold at 5 dai in syncytia formed by SCN in the compatible interaction with Williams 82 (Ithal et al. 2007), but does not change in expression in the incompatible interaction with Peking (Klink et al. 2007b) and with PI 88788 (Klink et al. 2010a, b). We overexpressed transcript Glyma08g11490.2, which was recently mapped and tested by complementation by Liu et al. (2012) to show that it confers resistance to SCN.

### Genes increasing the female index by more than twofold

Overexpression of several genes appeared to enhance susceptibility, including R30, C32, A21, and A61 (Table 3). Gene R30 (Glyma16g33840.1; EST BI972216) encodes an oligopeptide transporter that increased the SCN female index 2.5-fold when overexpressed in soybean roots. It has 96.7 % similarity at the amino acid level with Glyma09g29410.1 Transcripts of this gene were not altered in abundance on microarrays during the incompatible reaction with Peking, but were slightly down-regulated at −1.2 and −3.3-fold in the compatible interaction of SCN with soybean roots of Williams 82 at 2 and 10 dai, respectively (Ithal et al. 2007).

The gene C32 (Glyma02g37020.1; EST CF806679), encoding UDP-glucuronate 4-epimerase (EC 5.1.3.6), increased the female index 2.3-fold when overexpressed. Its closest relative at the aa level is Glyma17g07740.1 at 97.2 % similarity, with Glyma01g33650.1 and Glyma03g03180.1 having 77 % similarity. The transcripts of this gene were only slightly less abundant than controls in the incompatible reaction of SCN with Peking at 6 and 9 dai at −1.8 and −1.6-fold, respectively. Interestingly, this gene is increased 28.7-fold in galls formed by RKN in soybean roots two mai (Ibrahim et al. 2011).

Similarly, overexpression of gene A21 (Glyma15g03390.1; EST AW307334) that has no known function, yielded a 2.3-fold increase in the female index. This gene encodes a peptide of 134 aa. It possessed no domains similar to those in Pfam. It has similarity to Glyma13g41990.1 of 89.6 % at the aa level. Transcript levels of A21 were increased 16-, 31.3-, and 73.5-fold at 3, 6, and 9 dai in syncytia from the incompatible interaction of SCN with Peking (Klink et al. 2007a, b, 2009a). There was no change in the abundance of transcripts of A21 in syncytia of the compatible reaction in Williams 82 (Ithal et al. 2007).

The function of gene A61 (Glyma19g09810.1) is unknown, but the encoded protein contains a cupin domain. It is a redundant gene with six closely related homologs also on chromosome 19 and four other homologs with over 90 % similarity on chromosome 16. When this gene was overexpressed, the female index increased 2.1-fold compared to the control. In syncytia from the incompatible interaction of SCN with Peking, the abundance of the transcripts of this gene increased 23-, 87-, and 37-fold at 3, 6 and 9 dai, respectively (Klink et al. 2007a, b, 2009a). In contrast, transcript abundance in syncytia from the compatible interaction of SCN with Williams 82 only increased 3.8- to 5.3- fold at 2, 5 and 10 dai (Ithal et al. 2007).

Overexpression of the gene A32 (Glyma17g13550.1; EST CD397515), encoding pectate lyase, increased the female index of SCN almost two fold (1.97-fold). It has 93.6 % aa similarity with Glyma05g02890.1 and has more than 80 % aa similarity with six other soybean genes. It was overexpressed in syncytia 10- and 51-fold at 3 and 6 dai in the incompatible reaction with Peking and approximately 6.5-fold in the susceptible interaction with Williams 82 at 2 and 5 dai (Klink et al. 2007a, b, 2009a; Ithal et al. 2007).

### Other trends

**Fig. 4:**
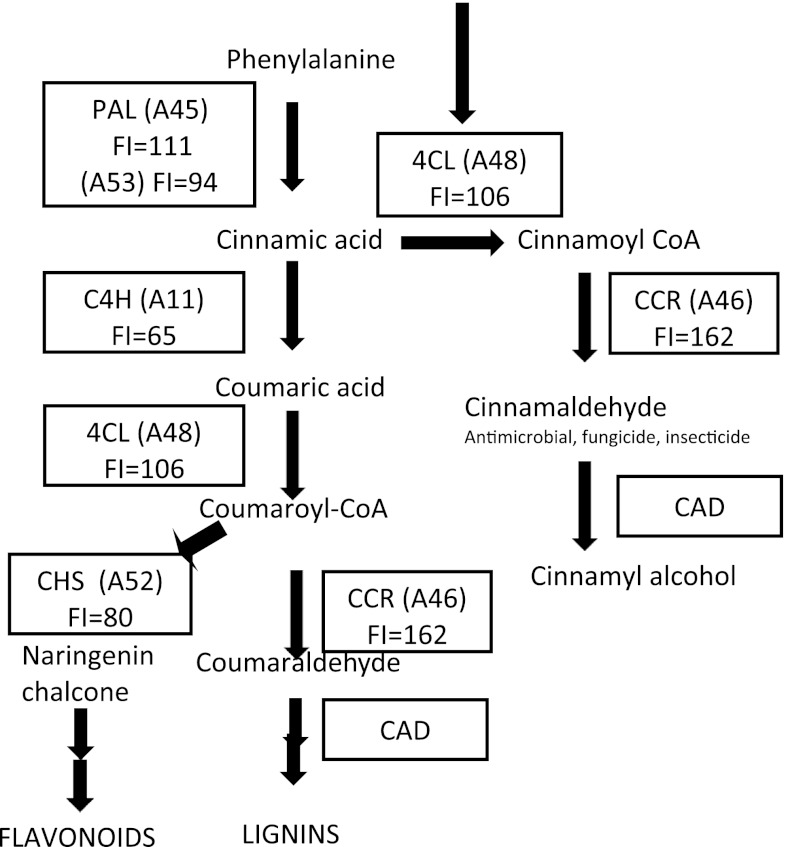
Simplified version of phenylpropanoid biosynthesis showing the location of tested genes encoding enzymes in the pathway. Genes tested included two encoding phenylalanine ammonia lyase (PAL, EC 4.3.1.24; A45, A53) and single genes encoding chalcone synthase (CHS, EC 2.3.1.74,; A52), 4-coumarate CoA ligase (4CL, EC 6.2.1.12, A48), cinnamate-4-hydroxylase (C4H, EC 1.14.13.11; A11) and cinnamoyl CoA reductase (CCR, EC 1.2.1.44; A46). Cinnamyl alcohol dehydrogenase (CAD, EC 1.1.1.185) is indicated, but was not tested

Flavonoids are derived from the phenylpropanoid pathway and carry out an array of functions (Samanta et al. 2011). Flavonoids accumulate in plant tissues upon infection by nematodes, and they are postulated as regulators of auxin transport (Murphy et al. 2000; Brown et al. 2001). Therefore, we examined the effect of overexpression of six genes related to flavonoid production, specifically, two genes encoding phenylalanine ammonia lyase (PAL, EC 4.3.1.24; A45, A53), and single genes encoding chalcone synthase (ChS, EC 2.3.1.74,; A52), 4-coumerate CoA ligase (4CL, EC 6.2.1.12, A48), cinnamate-4-hydroxylase (C4H, EC 1.14.13.11; A11), and cinnamoyl CoA reductase (CCR, EC 1.2.1.44; A46) (Fig. 4). Overexpression of genes encoding PAL (A45 and A53) had little effect on the female index of SCN in our assay (FI = 111 and 94, respectively), even though microarray analysis indicated that transcripts of both PAL genes (Glyma19g36620 and Glyma02g47940) were over 25-fold increased in abundance at 3, 6, and 9 dai in syncytia formed in the incompatible interaction of SCN with Peking (Klink et al. 2007b, 2009a, b). Both were also overexpressed in the incompatible interaction of SCN with PI 88788 (Klink et al. 2011), but there was no change in transcript abundance in the susceptible interaction at 2, 5, and 10 dai (Ithal et al. 2007).

Overexpression of the gene encoding C4H (A11) decreased the female index of SCN to 65 % of the control. Interestingly, the expression of this gene did not change at 3, 6, and 9 dai in syncytia of Peking and PI88788 exhibiting the incompatible interaction (Klink et al. 2009a, b, 2011). However, in syncytia of Williams 82 exhibiting the compatible interaction at 2, 5, and 10 dai, expression of the gene was 128-, 63-, and 107-fold increased (Ithal et al. 2007). Overexpression of the gene encoding 4CL (A48) had little effect on the female index of SCN (FI = 106). Expression of this gene was increased 21-, 29-, and 42- fold at 3, 6, and 9 dai in syncytia from Peking from the incompatible interaction (Klink et al. 2009a), but was unchanged at 2, 5, and 10 dai in syncytia from the compatible interaction (Ithal et al. 2007).

When the gene encoding CHS was overexpressed, the female index of SCN decreased by 20 %. The gene encoding CHS2 was overexpressed 47- and 40-fold at 3 and 9 dai, respectively, in syncytia of Peking exhibiting the compatible interaction (Klink et al. 2009a, b, 2011), while no change in gene expression was detected at 2, 5, and 10 dai in the compatible interaction with Williams 82 (Ithal et al. 2007). However, when the gene encoding CCR (A46) was overexpressed in soybean roots, the female index increased to 162 % of the control. Transcripts of the gene encoding this enzyme were increased 16-, 22-, and 44-fold in syncytia from the incompatible interaction of Peking at 3, 6, and 9 dai, respectively (Klink et al. 2009a).

Auxin is known to play a role in syncytium formation by the nematode (Grunewald et al. 2009a; Gheysen and Mitchum (2011). Therefore, we overexpressed three genes related to auxin to determine if they affected nematode development. One gene encoded an auxin repressor (R24); a second encoded an auxin permease (A18); the third encoded a WOX transcription factor (A06). Overexpression of the gene encoding the auxin repressor R24 had little effect on the female index (101 %). However, when the gene encoding auxin permease (A18) was overexpressed, the female index was increased to 161 %. The female index was increased to 139 % when the gene encoding the WOX transcription factor (A06) was overexpressed.

Because two auxin-related genes, A18 and A06, had an effect on SCN development, we examined the promoter region 2,000 bp 5′ to the ATG start site for each of the seven genes with the greatest positive effect on the female index (i.e., increased susceptibility to SCN). Promoters of five of these seven genes contained the ARF transcription factor binding sequence TGTCTC within 2,000 nt upstream of the ATG start site (Table 4). The promoter of gene A61 and C29 did not contain this transcription factor binding sequence. In contrast, when we examined the promoter region 2,000 bp 5′ to the ATG start site for each of ten genes with the greatest negative effect on the female index, none of these promoter regions contained the ARF transcription factor binding sequence.

**Table 4:** Occurrence of auxin response element TGTCTC in promoter of seven genes producing the highest female index of SCN when overexpressed in soybean roots

Gene ID no	Phytozome ID no	Predicted function	Location (nt)	Location on reverse complement (nt)	FI
R30	Glyma16g33840.1	Oligopeptide transporter	1,720, 253	–	254
C32	Glyma02g37020.1	UDP-glucuronate 4-epimerase	1,582	–	232
A21	Glyma15g03390.1	Unknown	1,426	–	229
A61	Glyma19g09810.1	Cupin domain	–	–	214
A32	Glyma17g13550.1	Pectate lyase	1,989	–	197
K01	Glyma15g41840.1	Lipase	–	–	195
A04	Glyma04g39860.1	Peroxidase III	1,685	148, 134	195

Location is number of nucleotides upstream from the ATG start site

In some cases at harvest, the females of SCN varied in size and color. The variation in color correlated with size such that smaller, less mature females were white, while larger, more mature females appeared yellow. The average number of mature females and small females were counted for four assays in which the genes A12, A25, A40, and A61 were overexpressed and the female index was greatly decreased (Table 5). The number of eggs was counted in each type of female. Fewer eggs were produced by females on the roots with low female index as compared to high female index. Thus, genes that decreased the female index greatly when overexpressed also produced females that produced fewer eggs.

**Table 5:** Effect of overexpression of genes on egg production

Construct	FI	Mean no mature females	Mean no small females	Mean no eggs/mature female	Mean no eggs/small female	Number of eggs
pRAP15 control	100	157	0.0	205	0.0	32,201
A12	36	37	5.6	138	12.2	5,244
A25	46	53	18.8	75	13.5	4,260
A40	71	89	22.4	94	18.4	8,806
A61	185	291	0.0	286	0.0	83,110

## Discussion

Plant parasitic nematodes establish a feeding site, in part, through effector proteins that the nematode injects into the selected host cell (Gao et al. 2003; Williamson and Kumar 2006; Caillaud et al. 2008; Klink et al. 2009b; Gheysen and Mitchum 2011; Haegeman et al. 2011). Analyses of these effector proteins, their localization within the host cell after secretion, and their interaction with host proteins provide insights into mechanisms involved in the formation of nematode feeding sites within a plant and provide clues to the molecular strategies used by the nematode to co-opt host cell functions. Global gene expression analysis has also contributed valuable information about host–pathogen interactions by measuring transcript abundance of genes during this battle for survival between a plant nematode and its host (Klink et al. 2007a, b, 2009a, 2010a, b; Ithal et al. 2007), while proteomics has provided insights into this interaction at the protein level (Afzal et al. 2009). Other approaches, including the use of RNA-Seq deep sequencing technologies promise to provide new insights to plant–nematode interactions (Matsye et al. 2011). While these approaches provide great new insights into host–nematode interactions, it is often difficult to interpret the data as to whether a gene or protein is produced by the host in its own defense or if it is expressed because the nematode has commandeered molecular machinery of the host to modulate gene and protein expression to provide a more favorable environment for nematode survival. As shown by this survey of the effect of overexpression of one hundred soybean genes in transgenic roots of composite plants on the female index of SCN, the actual effect of overexpression of individual genes on SCN can be determined, which helps in the interpretation of events occurring and underlying mechanisms during the host–pathogen interaction.

### Genes whose overexpression increase resistance

One gene that greatly decreased the female index of SCN when overexpressed in soybean roots was ascorbate peroxidase 2 (C21). Ascorbate peroxidase catalyzes the conversion of ascorbate and hydrogen peroxide to dehydorascorbate and water to detoxify hydrogen peroxide and helps to eliminate superoxide radicals and reduce apoptosis (Moon et al. 2002). Ascorbate plays an integral part in redox homeostatis, signaling, and peroxide metabolism. It is part of a redox hub that plays an important role in the response of plants to pathogens and in cell death (Foyer and Noctor 2011). We overexpressed two peroxidases in soybean roots, ascorbate peroxidase 2 (C21) and a cationic peroxidase (A02), and they had opposite effects. Overexpression of the first, ascorbate peroxidase 2 (C21), decreased the SCN female index more than 70 %, while the second (A02) increased the female index to 160 % of the control. *Arabidopsis* mutants exhibiting ascorbate deficiency produce microlesions, express higher levels of pathogenesis related protein 1 (PR1), and have increased basal resistance against *Pseudomonas syringae* (Kiddle et al. 2003; Pavet et al. 2005). Using the hydrogen peroxide responsive promoter of the *Ep5C* gene controlling the β-glucanase gene, Coego et al. (2005b) identified the *Arabidopsis* mutant *ocp3* (*overexpressor of cationic peroxidase 3*) which constitutively expresses *Ep5C.* These plants have increased resistance to the oomycete *Hyaloperonospora parasitica.* In our experiments, overexpression of ascorbate peroxidase 2 may deplete ascorbate levels, which trigger the defense response, similar to what was described in ascorbate-deficient *Arabidopsis* mutants. In contrast, overexpression of cationic peroxidase (A02) in soybean roots increased the female index of SCN. In tomato, a secreted cationic peroxidase confers susceptibility to *P. syringae,* and it is resistant to *P. syringae* when the expression of the *Ep5C* gene encoding the peroxidase is decreased by gene silencing (Coego et al. 2005a). Rather than providing resistance to biotrophic pathogens such as SCN, cationic peroxidase appears to contribute to resistance to necrotrophic pathogens. Our data indicate that these peroxidases play very different roles in the interaction between the host and the nematode, and that not all peroxidase genes increased in expression during nematode infection are providing resistance. In fact, some are supporting an environment enhancing nematode survival and development. Thus, although gene expression analysis provides broad insights into host–pathogen interaction, the over-expression of individual genes in the host, as shown here, can uncover specific details about gene function during the host–pathogen interaction.

Endo-β-1,4-glucanases hydrolyze 1,4-β-glycosidic linkages in cellulose. Overexpression of endo-β-1,4-glucanase (A12) decreased the female index of SCN to 40 % of the control. There is evidence that cell wall modifying enzymes can trigger plant defense responses to pathogens. Expression of an alfalfa β-1,3-glucanase gene in transgenic tobacco plants provided enhanced protection against the fungal pathogen *Cercospora nicotianae* or frogeye (Zhu et al. 1994). Expression of β-1,4 endo-xylanase from *Trichoderma reesei* in tall fescue resulted in necrotic lesions on the leaf suggesting that it can cause lesions similar to those triggered by ethylene and hydrogen peroxide (Buanafina et al. 2012). Recently, Hamamouch et al. (2011) showed that an *Arabidopsis* β-1,3-glucanase is the target of the cyst nematode effector protein 30C02 from *Heterodera schachtii* using yeast two-hybrid assays. When the β-1,3-glucanase *At4g16260* gene was overexpressed in *Arabidopsis*, the number of cysts per plant was decreased by approximately 22- to 38-percent. When the effector protein 30C02 was overexpressed in *Arabidopsis*, the number of cysts of *H. schachtii* per plant doubled. Furthermore, RNAi silencing of effector protein 30C02 decreased the average number of cysts per plant by approximately 75 %. Thus, overexpression of endo-β-1,4-glucanase may trigger a defense response in transgenic soybean that enhances resistance to SCN.

Momilactone A synthase is involved in diterpenoid phytoalexin synthesis. Momilactone A has only been found in rice and moss (Kato-Noguchi 2011). When we overexpressed momilactone A synthase (A24), the female index decreased to 41 % of the control. In soybean, portions of the pathway to the synthesis of momilactone A or similar compound may be present.

There is extensive evidence that the lipoxygenase gene family plays a role in resistance of plants to pathogens (e.g. Rance et al. 1998; Vijayan et al. 1998; For reviews see: Doke et al. 1996; Hammond-Kosack and Jones 1996; Feussner and Wasternack 2002). It is involved in the biosynthesis of jasmonic acid (Vick and Zimmerman 1983) and in signaling mechanisms associated with the plant defense response (Farmer and Ryan 1992; Bell and Mullet 1993; Berger et al. 1996; Review see Kazan and Manners 2008). Transcripts of lipoxygenase are increased 5- to 10-fold in *Arabidopsis* treated with methyl-jasmonate (Melan et al. 1993). Gao et al. (2008) showed that a maize *Mu*-insertional mutant of the 9-LOX gene, Z*mLOX3*, encoding 9-lipoxygenase, showed increased susceptibility to the root-knot nematode *M. incognita* with increased juveniles and eggs. Interestingly, the *lox3*-*4* mutants produced elevated levels of jasmonic and SA, yet they were still more susceptible to RKN. Our studies support a role for lipoxygenase in SCN resistance. Gene A25 encodes a protein containing a PLAT domain representative of lipoxygenase LH2; thus, it may play a role similar to lipoxygenase LH2-like proteins, which are associated with biosynthesis of certain plant defense compounds, such as phyto-oxylipins and jasmonic acid. When we over expressed gene A25 encoding a lipoxygenase in transgenic roots, there was a decrease in the female index of SCN.

Several genes of unknown function decreased the female index of SCN when overexpressed in roots. For example, C19, A30, and A08 all reduced the female index by 50 % or more when overexpressed. C19 and A30 have sequence similarity to cytochrome b5 proteins and plastocyanins, respectively; however, their functions remain to be determined.

The gene encoding serine hydroxymethyltransferase (Gene A35) is located on chromosome 8 (Glyma08g11490) of the soybean genome (Schmutz et al. 2010; www.Phytozome.com). Very recently, it has been shown to coincide with the *Rhg*4 locus conferring resistance to SCN (Liu et al. 2012) through mutation analysis, gene silencing and by transforming roots of a susceptible soybean recombinant inbred line, ExF63, with the gene to restore resistance. When we overexpressed this gene in susceptible roots, the female index of SCN decreased by 45 %. Microarray analysis of the expression of this gene indicates that the gene is expressed more in syncytia formed by SCN in Williams 82 in the compatible interaction as compared to cells from uninfected control roots. It is expressed at 3.1-fold at 2 dai and 2.1-fold at 5 dai (Ithal et al. 2007), but does not change in expression in the incompatible interaction with Peking (Klink et al. 2007b) nor with PI 88788 (Klink et al. 2010a). Matson and Williams (1965) first identified the *Rhg4* gene as a dominant gene required for resistance to SCN race 3. Rao-Arelli et al. (1992) demonstrated that Peking and PI 90763, resistant to SCN race 3, had three genes in common, one of which was *Rhg4*. The *Rhg4* gene was mapped to linkage group A2 using molecular markers (Weisemann et al. 1992; Matthews et al. 1998; Cregan et al. 1999; Qui et al. 2003); then it was physically mapped using BAC libraries by Lewers et al. 2001; 2002). TILLING experiments demonstrated that resistance is not conferred by the gene encoding an LRR-RLK that resides in the region (Liu et al. 2011). The historically defined SCN resistance loci had eluded identification for decades, and the gene expression experiments of syncytia undergoing the resistant reaction (Matsye et al. 2011) have provided a complimentary approach to fine mapping (Kim et al. 2010). For example, the expression of genes at the *rhg1* locus was examined by Matsye et al. (2011), and the gene encoding alpha soluble *N*-ethylmaleimide-sensitive factor attachment protein (α-SNAP; Glyma18g02590) that maps to the *rhg1* locus was recently examined for its effect on the female index of SCN (Matsye et al. 2012). Overexpression of that gene decreased the female index of SCN by approximately 50 % when overexpressed in soybean roots. The α-SNAP gene has been studied in numerous genetically tractable animal and fungal model systems (Weidman et al. 1989; Clary et al. 1990) and is part of the vesicular fusion and transport system that is involved in various types of plant defense responses (Collins et al. 2003; Assaad et al. 2004; Kalde et al. 2007; Patel and Dinesh-Kumar 2008; Hofius et al. 2009; Lenz et al. 2011; Lai et al. 2011).

Annexins have been implicated in the regulation of cell growth and in signaling during stress. A nematode effector protein was recently described from *H. schachtii*, and it is similar to annexins (Patel et al. 2010). This effector is a homolog of the *H. glycines* effector *Hg4F01* gene. The protein encoded by *Hs4F01* had 33 % identity with annexin-1 (*annAt1*) from *Arabidopsis*. Annexins are membrane and Ca2+ -binding proteins with many different functions (Talukdar et al. 2009). We overexpressed two genes encoding soybean annexin, Glyma13g27020 (C12) and Glyma13g27020 (A60) in soybean roots. Neither had a very significant effect on the female index of SCN when overexpressed, although C12 decreased the female index of SCN to 66 % of the control (*P* = 0.25). When the *H. schachtii* effector *Hg4F01* was overexpressed in *Arabidopsis*, the plants were more susceptible to *H. schachtii* and had approximately 25 % more cysts than wild-type plants (Patel et al. 2010). However, when *Arabidopsis* plants transformed with *Hg4F01* were infected with RKN, *M. incognita,* there was no significant change in the number of galls formed (Patel et al. 2010).

### Membranes and flavonoids

When the genes encoding several membrane proteins (including some transporters) were overexpressed, the female index of SCN changed dramatically. Some genes encoding membrane proteins, such as DREPP membrane protein (R8), aquaporin (A43), and a transporter (R48) decreased cyst development 34–50 %. In contrast, overexpression of other genes increased cyst development dramatically. These include genes encoding an oligopeptide transporter (R30), a metal ion transporter (C55), an auxin permease (A18), a membrane type III protein (A34), and a thaumatin-related protein (R04). The syncytium serves as a nutrient sink, providing resources for nematode growth and development. Several of these proteins may be involved in transport of nutrients to aid in the function of the syncytium. Auxin permease helps to regulate the auxin gradient within the plant and will be discussed below.

Flavonoids are produced in developing galls of RKN and in syncytia induced by the cyst nematode *H. schachtii* when infecting *Arabidopsis* (Jones et al. 2007). Mutant lines of *Arabidopsis* defective in portions of the pathway for flavonoid production supported nematode development, and there was no indication that flavonoids were required for syncytium development. In fact, Jones et al. (2007) suggested that flavonoids are produced by the plant as part of the defense response against nematodes. And Wuyts et al. (2006) showed that some flavonoids are toxic to nematodes. The role of flavonoids in defense against nematodes is supported by our data indicating that overexpression of several genes in flavonoid production influence the female index of SCN. We examined the effect of overexpression of six genes related to flavonoid production (Fig. 4). We overexpressed genes encoding two different phenylalanine ammonia lyases (PAL), the first enzyme in the pathway leading to phenylpropanoid production. However, neither PAL gene influenced the female index when overexpressed. This is in agreement with Wuyts et al. (2006), who showed that overexpression of PAL in *Arabidopsis* had no effect on RNK, *M. incognita,* reproduction. Overexpression of the gene encoding (4-hydroxy) cinnamoyl CoA (4CL) had little effect on the female index (106 %), while overexpression of cinnamoyl CoA reductase (CCR) increased the female index to 162 % of the control. CCR catalyzes reactions leading to guaiacyl and syringyl lignin production. It is uncertain why an increase in CCR expression leads to an increase in mature female nematodes, as this portion of the pathway leads to the synthesis of lignins, as well as many other compounds. Perhaps, overexpression of CRR is drawing carbon away from flavonoid production, therefore flow through that part of the pathway is reduced. This is supported in that overexpression of chalcone synthase (CHS) caused a modest decrease in the female index to 80 % of control. C4H converts cinnamic acid into p-coumaric acid, which is a precursor of both flavonoids and lignin. C4H decreased the female index to 65 % of the control. Thus, an increase in expression of C4H could increase availability of *p*-coumaric acid for increased flavonoid production supporting the host defense response.

### Influence of auxin in susceptibility

The level of indole-3-acetic acid or auxin in plant tissue is controlled though a variety of mechanisms, including synthesis, conjugation to amino acids (Ding et al. 2008), transcriptional repressors, influx, and efflux transporters (Grunewald et al. 2009a, b), and other means (Woodward and Bartel 2005). A role for auxin in the interactions of plant parasitic nematodes was suggested as long ago as 1948 by Goodey when describing galls formed by *Anguillulina balsamophila* on *Wyethia amplexicaulis* Nutt. leaves (Goodey 1948; for review see Grunewald et al. 2009a; Gutierrez et al. 2009; Kazan and Manners 2009). Balasubramanian and Rangaswami (1962) identified indole precursors of auxin in nematode galls of *Meloidogyne javanica.* When auxin (NAA) was applied to peach resistant to *M. javanica,* they became susceptible (Kochba and Samish 1971). IAA applied to tomato roots increased the size of galls formed by *M. javanica* (Glazer et al. 1986). Gruenwald et al. (2008) showed that the auxin-inducibe transcription factor AtWRKY23 was expressed during infection of *Arabidopsis* roots by *H. schachtii.* Recently, Grunewald et al. (2012) demonstrated that regulation of WRKY23 is controlled by the AUXIN RESPONSE FACTOR 7 (ARF7) and ARF19 pathway and that WRKY23 stimulates flavonol biosynthesis. Auxin had been shown to repress pathogenesis-related (PR) gene expression (Shinshi et al. 1987; Jouanneau et al. 1991). Extensive analysis of auxin mutants in *Arabidopsis* indicates that auxin signaling is antagonistic to and suppresses SA defense responses as reviewed in Kazan and Manners (2009).

Several lines of evidence from our data support a role for auxin in facilitating SCN development in soybean roots. Overexpression of a WOX transcription factor similar to WOX4 (WUSCHEL-RELATED HOMEOBOX4) increased the female index to 139 % of control. In *Arabidopsis*, the WOX4 transcription factor is required for auxin-dependent growth of cambium cells (Suer et al. 2011). The second gene that was overexpressed encoded an auxin-permease auxin influx-transporter protein1-like LAX 4, and it increased the female index to 161 % of control. Overexpression of the putative auxin repressor R24 had no effect on the female index. The protein encoded by R24 has 78 % amino acid identity to IAA16 [AT3G04730] from *Arabidopsis thaliana,* a repressor of auxin-responsive genes. The role of auxin in the enhancement of susceptibility within the host is emphasized further by the presence of the auxin transcription factor binding element ARF in the promoter region within 2,000 nt upstream of the start site of five of seven genes that increased susceptibility the most when overexpressed (Table 4). Combined, these facts confirm a role for auxin in plant susceptibility to nematodes.

### Functional genomics through candidate gene overexpression

Gene expression studies provide information as to what genes are increased and decreased in expression. However, during complex interactions between the host and a pathogen, such as a nematode, it is often unclear if an increase in gene expression is due to the host as a defense response to the pathogen or if it is due to the pathogen and its effector proteins and other substances produced to increase pathogen success. Testing one or two individual genes can be a project; testing the influence of a large number of host genes on their effect on resistance or susceptibility to specific pathogens can be a daunting task, because some plant species are difficult to genetically transform or it takes a long time to regenerate plants from callus or tissue culture. This is true in the case of soybean, because transformation and regeneration of whole plants can take 6–10 months and require sterile techniques.

The use of composite plants (Boisson-Dernier et al. 2001; Collier et al. 2005) dramatically reduces the time needed for testing genes. This approach has been particularly useful for soybean and its use in plant parasitic nematode research. Within 7 weeks of transformation, soybean roots are large enough to be challenged with nematodes (Klink et al. 2008, 2009b; Ibrahim et al. 2011). Composite soybean plants with transformed roots are limited in their usefulness in that they do not provide immortal lines through seed, and the inserted gene is not homozygous. However, composite soybean plants are very useful for a quick screen of many gene constructs to identify those genes worth testing further and do not require sterile technique. Notably, more than twelve interesting candidate genes chosen based on their gene expression patterns are shown to play important roles in host–nematode interactions. Several of these genes may be good candidates, alone and in combination for further testing to determine if the number of SCN females can be inhibited using whole transgenic plants.

## Abbreviations

ChS: Chalcone synthase
4CL: 4-hydroxy-cinnamoyl CoA
CCR: Cinnamoyl CoA reductase
Dai: Days after inoculation
eGFP: Enhanced green fluorescent protein
EST: Expressed sequence tag
FMV: Figwort mosaic virus
ORF: Open reading frame
PAL: Phenylalanine ammonia lyase
PCR: Polymerase chain reaction
RKN: Root-knot nematode
SCN: Soybean cyst nematode
